# A Cure for Wrinkles

**Published:** 1888-02

**Authors:** 


### A CURE FOR WRINKLES.

A curious application has been made of the absorbable properties of lanolin in the treatment of wrinkles. Although not strictly speaking a pathological condition, it is one which is even a more serious, because less avoidable, evil than freckles. When well rubbed in, lanolin passes directly into the skin, and acts as a nutrient to the subjacent tissues, with the effect of smoothing out the folds produced by the attenuation of these structures incidental to age. Several elderly ladies, who were induced to give this method of treatment a trial, are said to have been delighted with the result. —*Medical Press and Circular.*

